# How do virtual exercise technology and social presence influence motivation to exercise?

**DOI:** 10.3389/fpsyg.2026.1891596

**Published:** 2026-09-30

**Authors:** Sarina Sitsky, Rian H. Crowther, Vincent G. Kelly, Luke Wilkins, Tristan J. Coulter, Adam D. Gorman

**Affiliations:** 1 School of Psychology and Counselling, Faculty of Health, Queensland University of Technology, Brisbane, QLD, Australia; 2 QUT Sport, Queensland University of Technology, Brisbane, QLD, Australia; 3 School of Allied Health, Sport and Social Work, Griffith University, Gold Coast, QLD, Australia; 4 School of Exercise and Nutrition Sciences, Faculty of Health, Queensland University of Technology, Brisbane, QLD, Australia; 5 Centre for Sport and Human Performance Research, School of Allied Health, Human Services and Sport, La Trobe University, Melbourne, VIC, Australia

**Keywords:** attentional focus, competition, emotion, social comparison theory, young adults

## Abstract

**Introduction:**

Virtual technology offers a promising tool for encouraging engagement in exercise, as it can provide the user with enhanced visual stimuli and accessible metrics; however, the specific features within that technology that contribute to motivation and participation require further investigation. This study examined how different visual displays and the presence of a second rider influenced motivation to exercise using virtual technology.

**Methods:**

Twenty-four young adults completed a 3-km cycling course on the same virtual track under three counterbalanced conditions including (a) a metrics-only ride, where a screen in front of the rider displayed only standard performance variables (e.g., power, cadence), (b) a solo-virtual ride, which was identical to the previous condition, but with the addition of the participant being depicted on the screen as an avatar amongst virtual scenery, and (c) a paired-virtual ride, that contained the same elements as the previous two conditions, but with the addition of a second rider represented on the screen as an avatar.

**Results:**

The quantitative results, which included performance-related variables (cadence, power, speed, and heart rate), measures of intrinsic motivation, and ratings of perceived exertion, revealed significantly higher values for the paired-virtual condition compared to both the metrics-only and the solo-virtual conditions. Differences between the metrics-only and the solo-virtual conditions were isolated to interest-enjoyment and effort-importance. Brief interviews with participants indicated that the paired-virtual condition produced heightened levels of enjoyment and competitive drive which motivated participants to exert more effort. The solo-virtual condition was reported to be relaxing, but it was also reported as the least engaging. The metrics-only condition provoked negative emotions due to the absence of distractions and a heightened focus on internal cues.

**Discussion:**

The results highlight the psychological mechanisms that influence motivation and engagement when using virtual exercise technology.

## Introduction

Physical activity offers numerous benefits including improved cardiovascular health, and stronger bones, muscles, and joints (D’Onofrio et al., 2023). Recommendations from the World Health Organization (2020) suggest that people aged between 18 and 64 years should, at a minimum, complete between 150 and 300 min of moderate aerobic activity every week (or 75–150 min of vigorous aerobic activity per week). Despite the widespread benefits of physical activity (Warburton et al., 2006), evidence suggests that young adults do not always meet recommended physical activity guidelines (Abernethy et al., 2025; Australian Bureau of Statistics, 2023). This is concerning, as young people are potentially establishing physical activity habits that extend into later adulthood (Belanger et al., 2025). Investigating the factors that motivate young adults to engage in physical activity could therefore help to identify more effective strategies for promoting participation.

Virtual exercise technology may provide an effective strategy to motivate people to increase their levels of physical activity (Mouatt et al., 2020). Such technology can create an enjoyable exercise experience by replicating a real-world environment via a visual display, allowing the user to engage with the simulated surroundings (e.g., McDonough et al., 2020; Murray et al., 2016; Reason et al., 2025). One of the underlying mechanisms that appears to enhance the user experience when exercising with virtual technology is attentional focus (Baños et al., 2016; Mestre et al., 2011; Naugle et al., 2024; Neumann and Moffitt, 2018; see also Murray et al., 2016; Stewart et al., 2022). Specifically, such exercise tends to encourage the user to focus their attention on external factors such as the features within the visual display, which helps to distract from internal factors such as feelings of fatigue or discomfort (Baños et al., 2016; Mestre et al., 2011; Naugle et al., 2024). For instance, Mestre et al. (2011) showed that cycling on a stationary bicycle that displayed the rider as an avatar in a virtual display prompted participants to adopt an external focus of attention which was associated with lower levels of perceived exertion. The same task performed without the virtual environment produced the opposite effects with participants adopting a more internal attentional focus which was associated with higher levels of perceived exertion.

One of the other features of virtual exercise technology that may further influence motivation to exercise is the presence of other people in the simulated environment (Mestre et al., 2011; Michael and Lutteroth, 2020; Murray et al., 2016; Neumann and Moffitt, 2018; Nunes et al., 2014; Palmieri and Deutsch, 2024; Palmieri and Deutsch, 2025). Exercising in a virtual environment with a companion has been shown to increase levels of effort (Palmieri and Deutsch, 2024) and enjoyment (Mestre et al., 2011), and increase factors related to exercise intensity, such as heart rate (Murray et al., 2016), tension-pressure (Michael and Lutteroth, 2020), and ratings of perceived exertion (Nunes et al., 2014). Social comparison theory proposes that individuals will attempt to evaluate their own ability by using the performance of others as a benchmark (Festinger, 1954; Wood, 1989). Thus, when performing exercise alongside others, social comparison theory suggests that performers will be motivated to increase their levels of exertion to evaluate their performance relative to the other person (Festinger, 1954; Wood, 1989). However, as highlighted by Neumann and Moffitt (2018), a key limitation in the literature examining the presence of others in the virtual exercise environment is the use of instructions that either directly (e.g., Parton and Neumann, 2019; Snyder et al., 2012) or indirectly (Murray et al., 2016) encouraged participants to compete, thereby making it difficult to determine whether the participants were more motivated by the instructions or by the mere presence of the second person, or a combination of both.

Another limitation of the research examining virtual exercise technology is the use of experimenter-imposed exercise intensities that may have impacted intrinsic motivation and performance (Murray et al., 2016; e.g., Mestre et al., 2011; Neumann and Moffitt, 2018; Reason et al., 2025; Trewick et al., 2022). A prescribed intensity can negatively impact affective valence during exercise (Sheppard and Parfitt, 2008), and a fixed pace does not accurately reflect how people would exercise in real-world conditions when given the autonomy to self-select their exercise intensity to align with their capabilities. A further limitation is the lack of qualitative data to gain a deeper understanding of the factors that motivate people to engage in exercise involving the use of virtual exercise technology (but for exceptions, see Michael and Lutteroth, 2020; Nunes et al., 2014). Therefore, the use of a mixed methods research design to examine social presence without instructions that require a certain intensity or that encourage competition, alongside a comparable condition without social presence, is necessary to isolate and further explore the effects on motivation (Neumann and Moffitt, 2018; Nunes et al., 2014).

The present study used a mixed methods design where young adults were asked to complete the same 3-km ride on a virtual exercise device under three different conditions including (a) a metrics-only condition where a visual display showed performance variables that are typically displayed on a standard exercise bike, (b) a solo-virtual condition that included the same metrics as the previous condition, but with the addition of a visual display that depicted the participant as an avatar on a virtual track amongst surrounding scenery, and (c) a paired-virtual condition which was identical to the previous condition, but with the addition of a second rider who was visible on the track. The following hypotheses were generated:

*H1*: The paired-virtual condition would significantly increase participants’ motivation to exercise compared to the metrics-only condition, and this would be associated with higher levels of effort and exertion.

*H2*: The paired-virtual condition would significantly increase participants’ motivation to exercise compared to the solo-virtual condition, and this would be associated with higher levels of effort and exertion.

*H3*: The solo-virtual condition would significantly increase participants’ motivation to exercise compared to the metrics-only condition, and this would be associated with higher levels of effort and exertion.

## Methods

### Participants

A total of 24 participants (13 females; *M* age = 22.94 years; *SD* age = 1.69 years; age range = 18–24 years) were recruited for this study. A power analysis was conducted using G*power (version 3.1.9.7; Faul et al., 2007) for a one-way repeated measures ANOVA using within factors (effect size *f =* 0.25; correlation = 0.6; alpha = 0.05). The analysis indicated that a minimum sample size of 23 participants was required to achieve a statistical power of 0.8. All participants received a Coles eGift Card to the value of $50 (AUD). In alignment with the definition of young adults used by The Society for Adolescent Health and Medicine (Walker-Harding et al., 2017), participants could be recruited if they were aged between 18 and 25 years (the actual range for the present study was 18 to 24 years). The study received institutional ethics approval, and all participants provided informed consent.

### Equipment

A Zwift ride smart stationary bike was coupled with a 74-inch Samsung flat screen TV for all experimental conditions (for images of the typical display used in the Zwift system, see https://support.zwift.com/en_us/using-the-heads-up-display-when-cycling-rJepA4beS). In two of those experimental conditions (solo-virtual and paired-virtual), the TV displayed a virtual image of a pre-determined cycling track (Volcano Flat) from the Zwift system, with the participants shown as an avatar riding a bicycle. Inclines and declines were simulated on the Zwift bike whenever participants were riding up or down a hill. A Wahoo KICKR (Wahoo, 2026) was attached to the base of the Zwift bike to measure speed (km/h), distance (km), power (watts), and cadence (revolutions per minute; RPM).

### Quantitative measures

#### Intrinsic motivation inventory

The intrinsic motivation inventory is a scale used to measure intrinsic motivation (defined as a person’s internal desire to engage in an activity to gain pleasure and satisfaction; Weinberg and Gould, 2011) and is based upon the central components of self-determination theory (Ryan, 1982). It has been widely used across a range of domains including education (Cocca et al., 2022), sport (Grech et al., 2024), and healthcare (Triebner et al., 2024). The present study used McAuley et al.’s (1989) shortened scale, however this was modified to include only four subscales including interest-enjoyment, effort-importance, tension-pressure, and competence, which were most relevant to the present study (Table 1). McAuley et al. (1989) reported good internal consistency among the items in each subscale. Item wordings were slightly modified by the research team to suit the nature of the study. For example, an item from the interest-enjoyment subscale was changed from *‘I enjoyed this basketball game very much’ to ‘I enjoyed this activity very much’.* All items were measured on a 7-point Likert scale where participants reported how true each statement was for them with 1 indicating ‘not at all’ and 7 indicating ‘very true’. Reverse-scored items were recoded, and the average scores for each subscale were calculated.

**Table 1:** Intrinsic motivation inventory subscale items and Cronbach’s alpha levels.

Intrinsic subscale	Number of initial items	Number of items remaining	Cronbach’s alpha
Interest-enjoyment	5	4	0.87
Competence	3	2	0.79
Effort-importance	4	3	0.92
Tension-pressure	4	3	0.76

#### Ratings of perceived exertion

The Borg (1982) ratings of perceived exertion (RPE) scale was used to determine participants’ subjective perceived effort. The scale ranged from 6 to 20 where 6 indicated no exertion levels, and 20 indicated maximal exertion. The Borg RPE is a valid and reliable tool that can be used with different populations (Grummt et al., 2024; Lea et al., 2022). A systematic review and meta-analysis including 118 studies found the overall mean validity coefficient for the scale was 0.88 (Lea et al., 2022). Scherr et al. (2013) reported that the tool had a strong criterion validity and showed high correlations with heart rate and blood lactate.

#### Performance measures

Power (watts) and cadence (RPM) were measured and collected by the Wahoo KICKR (Wahoo, 2026). A bluetooth heart rate monitor (Garmin HRM-Pro) was worn by all participants to record their heart rate in beats per minute, and this information was displayed on the screen. Power, cadence, and heart rate data were downloaded from the Zwift system for later analysis. The sampling rate of the Zwift system was 1 Hz, and this rate applied to all performance measures.

### Procedure

Participants completed the experiment in an indoor cycling studio that contained screens that were connected to Zwift bikes. Before the study, participants completed the adult pre-exercise screening system to ensure they had no pre-existing health conditions or injuries that would pose a risk during the experiment (Exercise and Sports Science Australia, 2024). Participants were not regular cyclists and reported no prior experience using virtual cycling technology. The exclusion of participants who were cyclists or who had experience using virtual cycling technology was implemented to control for prior experience effects and to standardise participant characteristics. Participants were advised to abstain from alcohol and caffeinated beverages in the 24 h prior to the experiment, as this may have affected their perceived exertion. The experiment took approximately 60 min per participant to complete. Two participants completed the experiment at the same time, and whilst each participant was aware that another participant was in the room, they were separated by a large divider that prevented them from seeing each other. Each pair of participants was randomly allocated to a counterbalanced order to minimise the potential influence of order effects. This setup equated to six different orders with four participants allocated to each order, although due to a technical issue during a testing session, one of those orders had six participants (metrics-only; paired-virtual; solo-virtual) and another had two participants (metrics-only; solo-virtual; paired-virtual).

On arrival, participants were provided with a detailed overview of the study, and they were asked to verbally confirm their consent to complete the experiment. To ensure minimal influence over affective valence (see Sheppard and Parfitt, 2008), the lead researcher asked participants to ride the bike at their self-selected pace until they reached the end of the 3-km course, and no further instructions were provided. To avoid influencing participants’ level of exertion, no emphasis was placed upon intensity, speed, or competition. If participants enquired about these elements, the researcher simply reminded participants to self-select their pace. The researcher adjusted the bike height to ensure optimal comfort for the participant. A profile was then made for the participant, entering their date of birth, height, and weight into the Zwift system. Height was measured with a stadiometer and weight was measured using a digital scale. The participant was then asked to sit on the bike, and they were shown how to change gears. A 5-min self-paced ride was then completed to ensure the participant was confident in changing gears, and to also provide a warm-up before the first condition.

In the *metrics-only condition*, the majority of the screen in front of the participant was covered by a black cloth, though basic performance metrics remained in view, including speed, power, average power, kilojoules burnt, cadence, heart rate, duration, and distance covered. This condition was designed to replicate a traditional exercise bike that did not display virtual features. In the *solo-virtual condition*, participants could view the same performance data as in the metrics-only condition, however the black cloth was removed to reveal a virtual track and an avatar of the rider that mimicked the pedal movements of the participant. The back of the avatar was visible from a slightly elevated angle, thus providing a third-person view. This condition provided an opportunity to determine whether the motivation and performance of the participant was influenced by the addition of virtual visual information. In the *paired-virtual condition*, the same visual information from the solo-virtual condition was displayed, but with the addition of a second rider (the other participant in the room) who was shown as another avatar on the track. Both participants were informed that the second avatar was the other participant in the room, but they were not aware that the “rubber-band” feature had been enabled in the Zwift system. This feature meant that both participants would remain visible on each of their respective screens, regardless of their actual speeds. The dynamic adjustments ensured that the two riders stayed in close virtual proximity despite differences in their cycling output – it did not place the second avatar at a fixed distance and relative position could change (i.e., the participant could be in front of or behind the second avatar). This rubber-banding feature not only mitigated the potential differences in the cycling capabilities of each pair of participants, but it also ensured the paired-virtual condition displayed the second rider for the full duration of the ride. The constant presence of the second rider was important because if one of the two riders obtained a substantial lead during the ride, then the influence of the second rider could have had a diminished and variable impact on participants’ motivation and performance. None of the participants reported that they were aware of the rubber-banding feature, and there were no statements made by the researcher that encouraged the participants to compete with the second rider. The paired-virtual condition therefore provided an opportunity to determine whether the motivation and performance of participants were influenced by the addition of a second rider on the track.

After completing each 3-km condition, participants were immediately asked to complete the RPE scale and the intrinsic motivation inventory scale. After the three conditions were completed, each participant took part in a brief interview which was led by the first author. The questions were designed to gather additional details about the participant’s experiences (e.g., “briefly describe your emotions for each condition and explain why you felt those emotions” and “if you could choose only one of the three conditions to use over a longer period of time, which condition would you choose and why?”), their attentional focus (“what were you mostly focusing your attention on during each condition?”), and their intentions regarding the possibility of using virtual exercise technology in the future (e.g., “overall, what would motivate you to continue using an indoor exercise bike?” and “would you like to continue to use an indoor cycling system like the Zwift, and if so, what aspects appeal to you the most?”).

### Data analysis

#### Quantitative analysis

One-way repeated measures ANOVAs were used to analyse each dependant variable and examine the differences across the three conditions. The same analyses were also used to examine whether there were any testing order effects (i.e., first condition, second condition, third condition). Sidak adjustments were applied to each post-hoc comparison to reduce the chance of Type 1 errors. A reliability analysis was conducted on each item of the shortened intrinsic motivation inventory to determine intercorrelations between item-scale and identify any potentially redundant items. Following the guidelines of Field (2014), four items were removed across the subscales due to their item-total correlations being below 0.3. Table 1 shows the number of items in each revised scale, and their overall Cronbach’s alpha after items were removed.

Assumptions of normality were checked using Tabachnick and Fidell’s (2001) method of comparing skewness and kurtosis statistics to their standard errors to create z-scores. Interest-enjoyment in the metrics-only condition and heart rate in the paired-virtual condition showed deviations from normality. Similarly, for the data used to examine the testing order effects, heart rate for the first condition showed deviations from normality. Friedman’s tests were conducted to determine whether a non-parametric test would produce different results to those generated by the one-way repeated measures ANOVAs. The results from Friedman’s tests for the non-normal data produced similar results to the ANOVAs, with interest-enjoyment_metrics-only_ (χ^2^ = 30.02, *p* = <0.001) and heart rate_paired-virtual_ (χ^2^ = 9.08, *p* = 0.011) remaining significant, and with heart rate_first_condition_ (χ^2^ = 5.25, *p* = 0.072) remaining non-significant. Therefore, to maintain consistency, only the results from the ANOVA are reported. To address the assumption of sphericity, Greenhouse-Geissier adjustments were applied to heart rate, power, and competence for the ANOVAs in the main analyses. The same adjustments were applied for heart rate and competence for the ANOVAs used for the testing order effects. Alpha was set at 0.05 for all statistical analyses.

#### Qualitative analysis

The brief interviews (*M* duration = 5 min 54 s) were thematically analysed, as thematic analysis is a robust and widely used psychological method for identifying and interpreting themes in qualitative data (Ahmed et al., 2025). An interpretivist epistemological stance was used to analyse the data because this acknowledges how participants’ experiences are subjective and socially constructed (Bunniss and Kelly, 2010; Daher et al., 2017; Thanh and Thanh, 2015). This stance allowed the researcher to focus solely on the meanings of the participants’ responses, rather than seeking objective truths (Bunniss and Kelly, 2010; Thanh and Thanh, 2015). Following the thematic guidelines proposed by Braun and Clarke (2006), the lead researcher manually transcribed participants’ responses, and then repeatedly read those responses to search for any pattern prior to initial coding. Codes were generated and then grouped into higher-order themes, lower-order themes, and sub-themes. These codes were subject to ongoing refinement by engaging with other members of the research team to offer alternative explanations. For example, the original higher-order themes of *“attention and affect”, “perceived performance”,* and “*competitiveness”* were changed to lower order themes with a refined higher order theme of “*psychological constructs.”*

## Results

### Quantitative results

The means and standard deviations for the quantitative data are shown in Table 2.

**Table 2:** Descriptive data for intrinsic motivation, RPE, power, cadence, and heart rate.

	Paired-virtual	Solo-virtual	Metrics-only
Measures	M (SD)	M (SD)	M (SD)
Interest-enjoyment	4.71 (1.16)	3.47 (0.95)	2.35 (1.24)
Competence	4.42 (1.25)	3.88 (1.13)	3.54 (1.54)
Effort-importance	5.94 (0.78)	4.10 (0.98)	3.39 (1.41)
Tension-pressure	4.38 (1.43)	2.78 (1.08)	2.25 (1.02)
RPE	15.70 (2.35)	13.60 (2.35)	12.80 (1.79)
Power (Watts)	150.00 (54.27)	114.00 (42.9)	112.00 (37.10)
Cadence (RPM)	88.20 (10.18)	80.50 (9.18)	80.90 (10.90)
Heart rate (BPM)	165.00 (23.52)	151.00 (16.33)	147.00 (19.10)

M = mean; SD = standard deviation; RPE = rating of perceived exertion; RPM = revolutions per minute; BPM = beats per minute.

The one-way repeated measures ANOVAs used to analyse the quantitative data revealed significant differences (*p* < 0.05) for all dependent variables (Table 3). Effect sizes ranged from 0.21 to 0.65, constituting large effect sizes (Cohen, 1988). The one-way repeated measures ANOVAs that were used to examine testing order effects produced non-significant results (*p*-values ranged between 0.433 and 0.930), suggesting that the data from the main analyses were not influenced by the order in which participants completed the experimental conditions.

**Table 3:** Results for the one-way repeated measures ANOVAs used to analyse the quantitative data.

Measure	F (df)	*p*	η^2^_p_
Interest-enjoyment	38.75 (2, 46)	<0.001	0.63
Competence	6.25 (1.33, 30.56)	0.012	0.21
Effort-importance	42.01 (2, 46)	<0.001	0.65
Tension-pressure	38.61 (2, 46)	<0.001	0.63
RPE	11.94 (2, 46)	<0.001	0.34
Power	21.30 (1.50, 34.46)	<0.001	0.48
Cadence	11.17 (2, 46)	<0.001	0.33
Heart rate	7.48 (1.54, 35.32)	0.004	0.25

For hypothesis 1, all pairwise comparisons between the paired-virtual and metrics-only conditions were significant (Table 4). Specifically, the results for all quantitative variables were significantly higher in the paired-virtual condition, indicating that participants reported greater motivation and exertion, and displayed greater physiological responses when cycling in the paired-virtual condition compared to the metrics-only condition. For hypothesis 2, all pairwise comparisons between the paired-virtual and solo-virtual conditions were also significant. Again, the results for all quantitative variables were significantly higher in the paired-virtual condition, indicating that participants reported greater motivation and exertion, and displayed greater physiological responses when cycling in the paired-virtual condition compared to the solo-virtual condition. For hypothesis 3, six of the eight pairwise comparisons between the metrics-only and solo-virtual condition were non-significant. The two variables for which significant differences were found were the interest-enjoyment and effort-importance subscales from the intrinsic motivation inventory. Here, participants reported significantly higher levels of interest-enjoyment and effort-importance for the solo-virtual condition, indicating greater motivation when cycling in the solo-virtual condition compared to the metrics-only condition.

**Table 4:** Post-hoc results for all quantitative data.

Measure	Comparison	95% CI	*p*	*d*
Interest-enjoyment	Metrics-only - Paired-virtual	−3.18, −1.52	<0.001	−1.49
	Paired-virtual - Solo-virtual	0.62, 1.86	<0.001	1.05
	Metrics-only - Solo-virtual	−1.71, −0.52	<0.001	−0.99
Competence	Metrics-only - Paired-virtual	−1.71, −0.04	0.037	−0.55
	Paired-virtual - Solo-virtual	0.10, 0.98	0.012	0.65
	Metrics-only - Solo-virtual	−0.93, 0.27	0.418	−0.29
Effort-importance	Metrics-only - Paired-virtual	−3.44, −1.67	<0.001	−1.52
	Paired-virtual - Solo-virtual	1.23, 2.47	<0.001	1.57
	Metrics-only - Solo-virtual	−1.41, −0.10	0.046	−0.53
Tension-pressure	Metrics-only - Paired-virtual	−2.89, −1.37	<0.001	−1.47
	Paired-virtual - Solo-virtual	0.98, 2.21	<0.001	1.36
	Metrics-only - Solo-virtual	−1.08, 0.02	0.064	−0.50
RPE	Metrics-only - Paired-virtual	−4.37, −1.29	<0.001	−0.98
	Paired-virtual - Solo-virtual	0.47, 3.70	0.009	0.68
	Metrics-only - Solo-virtual	−2.23, 0.73	0.498	−0.27
Power	Metrics-only - Paired-virtual	−56.40, −19.50	<0.001	−1.08
	Paired-virtual - Solo-virtual	15.78, 54.60	<0.001	0.95
	Metrics-only - Solo-virtual	−13.66, 8.14	0.890	−0.13
Cadence	Metrics-only - Paired-virtual	−12.51, −2.06	0.005	−0.73
	Paired-virtual - Solo-virtual	2.49, 12.89	0.003	0.78
	Metrics-only - Solo-virtual	−3.11, 3.92	0.988	0.06
Heart Rate	Metrics-only - Paired-virtual	−32.19, −3.19	0.014	−0.64
	Paired-virtual - Solo-virtual	0.63, 28.00	0.038	0.55
	Metrics-only - Solo-virtual	−11.83, 5.07	0.677	−0.21

### Qualitative results

Thematic analyses of the qualitative data revealed three higher-order themes: *psychological construct, condition preference,* and *factors influencing motivation* (Table 5).

**Table 5:** Results from the thematic analysis.

Higher-order	Lower-order	Sub-themes
Psychological construct (23)	Competitiveness (19)	External competitiveness. Internal competitiveness. Non-competitive.
	Perceived exertion (14)	Higher perceived exertion to beat the second rider. Lower perceived exertion due to no second rider. Higher perceived exertion to complete task quicker.
	Attention and affect (10)	Negative affect due to internal focus. Positive affect due to external focus.
Condition preference (23)	Paired-virtual (14)	Competitive enjoyment.
	Solo-virtual (7)	Screen provides entertainment. No competitive pressure.
	Metrics-only (2)	Greater internal focus.
Factors influencing motivation (18)	Social aspect (9)	Peer-driven motivation.
	Outdoor enjoyment (6)	Environmental enjoyment. Can speak to someone while exercising.
	Accessibility (5)	Willingness to use if easily accessible.
	Perceived choice and autonomy (4)	The variety of different routes maintains interest.

Total number of participants contributing to each higher- and lower-order theme are shown in parentheses.

#### Psychological construct

The first higher-order theme reflects participants’ psychological thought processes, and how these impacted their motivation towards virtual exercise technology. The first lower order theme, *competitiveness,* captured participants’ motivation to achieve their goals. It consisted of three sub-themes including *external competitiveness, internal competitiveness,* and *non-competitiveness. External competitiveness* was evident in the paired-virtual condition and refers to how the participants focused solely on beating the second rider, whereas *internal competitiveness* emerged mostly from the solo-virtual ride and reflects participants’ desire to surpass their performance on previous conditions and to achieve their personal goals. *Non-competitiveness* was evident in all conditions and represents participants who were not motivated by a desire to beat the second rider, nor were they motivated to achieve certain performance outcomes (e.g., time to complete the 3-km course). “I felt even if I did win, I didn’t win anything, so I didn’t really try (P8)”.

The second lower order theme, *perceived exertion*, consists of three sub-themes, including *higher perceived exertion to beat the second rider, lower perceived exertion due to no second rider,* and *higher perceived exertion to complete the task quicker.* In the paired-virtual condition, participants reported higher levels of motivation and exertion, which were reportedly driven by their desires to beat the second rider. In the conditions without a second rider, motivation and exertion decreased, with participants reporting that they had no incentive to ride faster. In the metrics-only condition, participants’ determination to finish the ride motivated them to exert more effort which was exemplified by the following quote: “The last one [metrics-only condition] I tried a little harder so it would finish sooner because I was really bored (P16)”. This quote suggests that in the absence of virtual features and a second rider, some participants increased their exertion out of a desire to complete the task quicker, indicating that motivational focus may shift from performance-orientated to goal-termination motives when engagement is low.

The last lower order theme, *attention and affect*, describes participants’ focus and how this impacted their emotional state and, in turn, their motivation. The first sub-theme of *negative affect due to internal focus* was evident during the metrics-only condition. Participants reported that due to the limited distractions in the metrics-only condition, their focus shifted internally, and the internal focus decreased their mood and increased their awareness of the fatigue their body was experiencing. “The second one [metrics-only condition] was boring, very boring. I had nothing to focus on other than my sore legs, so I didn’t enjoy that one (P3).” The second sub-theme, *positive affect due to external focus*, emerged from the solo-virtual and paired-virtual conditions. Participants reported an external focus of attention in both conditions and highlighted how they experienced greater levels of enjoyment and were more engaged with the activity which enhanced their motivation and sustained their effort. Participants reported the solo-virtual condition to be peaceful and relaxing as they were able to ride at their own pace while still having the distraction of the visual elements displayed on the screen. “It was nice just being able to set my own pace, not trying to stay close to another person but still having the screen to keep me interested” (P18).

#### Condition preference

When participants were asked about the condition they enjoyed most, the *paired-virtual* condition was most commonly selected (*n* = 14), followed by the *solo-virtual* condition (*n* = 7), and then the *metrics-only* condition (*n* = 2) (one participant did not have a preference).

The paired-virtual condition was preferred due to participants finding it more enjoyable to compete against someone, as opposed to riding solo, which formed the sub-theme of *competitive enjoyment.* However, some participants reported how they preferred the *solo-virtual* condition, primarily because they believed they would not be able to sustain the exertion levels that were required of them in the paired-virtual condition. Instead, they enjoyed the element of relaxation and peacefulness throughout the ride in the solo-virtual condition, mostly because they did not need to race against or maintain their pace with a second rider. This formed the sub-theme of *no competitive pressure.* Another sub-theme that was developed from the *solo-virtual* condition was *screen as a distraction*, as participants reported how the screen shifted their attention to external factors. Participants who preferred the *metrics-only* condition felt as though they were better able to internalise their physiological and emotional states without having external distractions. This formed the sub-theme of *greater internal focus*. “I was focusing on how I was controlling breathing and regulating everything because there was nothing to focus on except that” (P24).

#### Factors influencing motivation

The third higher order theme, *factors influencing motivation,* consisted of factors that affected participants’ motivation to continue using an indoor virtual exercise bike such as the Zwift system. These factors formed four lower order themes, namely *social aspect, outdoor enjoyment, accessibility,* and *perceived choice and autonomy.*

*Social aspect* explained how the presence of another rider on the screen increased participants’ motivation to exercise. Participants reported that exercising alongside another person helped to sustain their motivation, which formed the sub-theme of *peer-driven motivation*. The lower order theme of *outdoor enjoyment* was a factor that decreased participants’ motivation to ride indoors, if outdoor riding was an option. Relatedly, the sub-theme of *environmental enjoyment* highlighted how participants enjoyed the environmental aspects of riding outdoors, such as experiencing the sunshine, fresh air, and the “feeling of going fast (P9)” when riding outdoors. The lower-order theme *accessibility* was described by participants as something that would decrease their motivation to use an indoor system, such as Zwift. The participants expressed that they would use an indoor virtual exercise bike if it was easily accessible, but they also reported a lack of motivation to travel long distances to use such a system, and this formed the sub-theme of *willingness to use if easily accessible.* Finally, the lower-order theme of *perceived choice and autonomy,* with the sub-theme of *a variety of different routes maintains interest,* highlighted how participants wanted the option to change the scenery on each ride to help motivate them to continue using a virtual indoor exercise bike.

## Discussion

This study used a mixed methods research design to explore the factors that motivate young adults to perform exercise using virtual technology. The results relating to each of the three hypotheses are discussed first, followed by a discussion of the other factors that influenced motivation. Limitations and future directions are also discussed.

### Hypothesis one - paired-virtual condition versus metrics-only condition

For the first hypothesis, it was predicted that the paired-virtual condition would significantly increase participants’ motivation to exercise compared to the metrics-only condition, and this would be associated with higher levels of effort and exertion. Consistent with this hypothesis, the paired-virtual condition showed significantly higher levels of motivation in the form of interest-enjoyment, effort-importance, and competence compared to the metrics-only condition. There were also significantly higher values for exertion with tension-pressure, RPE, heart rate, power, and cadence increasing in the paired-virtual condition compared to the metrics-only condition. An explanation for these findings can be gleaned from the qualitative data where participants reported that the presence of the second rider in the paired-virtual condition motivated them to compete with the other rider. In alignment with social comparison theory (Festinger, 1954; Wood, 1989), it is possible that the participants’ competitive drive was piqued by their desire to evaluate their ability against the performance of the other rider on the track. These findings are also consistent with a systematic review that found virtual avatars to be an effective way to boost engagement and motivation during exercise (Mouatt et al., 2020). Similarly, Murray et al. (2016) reported participants had a higher heart rate and covered a longer distance when rowing with a companion in a virtual reality condition compared to rowing without a virtual companion, and Mestre et al. (2011) showed that riding with a partner in a virtual reality condition significantly increased enjoyment compared to riding with no virtual reality. However, the present results extend those reported previously by showing that a more neutral instructional set, that is, instructions that do not specifically encourage participants to focus on beating the other rider (e.g., Parton and Neumann, 2019) or to maintain a certain pace (e.g., Mestre et al., 2011), will tend to naturally create a competitive environment that may increase effort as well as the motivation to exercise (see also Milyavskaya et al., 2021; Nunes et al., 2014).

The significantly higher levels of competence in the paired-virtual condition, compared to the metrics-only condition, suggests that the addition of a virtual stimulus and the presence of a second rider can increase intrinsic motivation. According to the theory of self-determination, higher competence enhances intrinsic motivation as individuals who feel capable of participating in an activity are more engaged and willing to exert effort (Ryan and Deci, 2020). Although it was not mentioned specifically by participants during the interviews, it is possible that the higher perception of competence was influenced by the Zwift banding feature, as participants likely perceived themselves as more competent due to maintaining their position alongside the second rider on the screen. The close proximity of the second rider (created by the banding feature in the Zwift system) may have functioned as a salient benchmark that participants could use to evaluate their performance, thereby further contributing to their higher levels of exertion (see Festinger, 1954; Wood, 1989).

During the interviews, participants also described how the presence of the second rider in the paired-virtual condition created an external focus of attention, which has been shown in previous research to help reduce feelings of exertion (Mestre et al., 2011; Naugle et al., 2024). That is, when individuals exercise using virtual stimuli, they tend to direct their attention externally, distracting them from their internal cues and lowering their perceived exertion relative to their actual effort (Mestre et al., 2011; Naugle et al., 2024). However, despite reporting the use of an external focus, participants in the present study reported significantly higher levels of exertion (RPE) during the paired-virtual condition. It is therefore likely that the participants’ motivation to beat the other rider may have limited the extent to which the external focus could reduce their feelings of exertion. In other words, the increased intensity required to compete with the other rider likely diminished the benefits of an external focus of attention created by the virtual display.

In contrast to the generally positive outcomes associated with the paired-virtual condition (and the solo-virtual condition), participants reported in their interviews that the metrics-only condition was boring and unstimulating, which made it difficult to engage with the activity. The qualitative data also showed that the metrics-only condition created a negative affect for some participants because it encouraged them to adopt an internal focus of attention. These findings further highlight how participants’ levels of enjoyment for a given activity can be increased by including virtual external stimuli that serve as a distraction from their internal sensations (see Baños et al., 2016; Mestre et al., 2011; Murray et al., 2016; Neumann et al., 2018).

### Hypothesis two - paired-virtual condition versus solo-virtual condition

For the second hypothesis, it was predicted that the paired-virtual condition would significantly increase participants’ motivation to exercise compared to the solo-virtual condition. In a similar vein to the results reported for the first hypothesis, participants had higher levels of exertion as demonstrated by significantly higher values for tension-pressure, power, heart rate, cadence, and RPE in the paired-virtual condition compared to the solo-virtual condition. Participants were also more motivated in the paired-virtual condition with significantly higher ratings of interest-enjoyment, effort-importance, and competence compared to those found for the solo-virtual condition. These results align with Nunes et al. (2014) who found that exercising on a treadmill while viewing a virtual image on a screen was more motivating for participants when they were in a competitive scenario, and it also produced higher RPEs and heart rates compared to when they used the same virtual device on their own (for examples of other research that examined the influence of competition, see Palmieri and Deutsch, 2024; Palmieri and Deutsch, 2025). However, while Murray et al. (2016) found similar results in the form of significantly higher values for heart rate and the amount of distance covered when participants performed rowing with a partner in a virtual environment compared to rowing alone in that same environment, their results also showed no significant differences in several other measures including power, RPE, enjoyment, and intrinsic motivation. Variations in the results between the present study and those reported by Murray et al. (2016) are most likely explained by differences in the instructional sets. Specifically, the instructions provided by Murray et al. (2016) likely encouraged competition, whereas those used in the present study were more neutral in nature and were not intended to directly or indirectly encourage participants to compete. Further research should be conducted to explore the underlying factors that influence exercise intensity and motivation when performing in the presence of others, with a particular focus on the influence of different instructional sets (see Neumann and Moffitt, 2018).

Despite the increased levels of enjoyment and the higher levels of physical exertion exhibited for the paired-virtual condition, participants reported that they would not be able to sustain such a high intensity over an extended duration. This highlights the role of exertion in shaping motivation, as when individuals find an exercise overly fatiguing or beyond their capabilities, the taxing effect on the body may undermine motivation and create a situation where individuals may be less inclined to participate (Parfitt et al., 2015; Park et al., 2012; Parton and Neumann, 2019). Ultimately, it suggests that enjoyment is not a sole predictor of motivation (Lemmens, 2023), and the extent to which the individual feels that the activity is achievable will affect their motivation to continue over time (Parfitt et al., 2015; Parton and Neumann, 2019).

Finally, the significantly higher levels of perceived competence for the paired-virtual condition compared to the solo-virtual condition can be explained in an identical manner to those described for hypothesis one. That is, the banding feature that was used in the Zwift system during the paired-virtual condition, which ensured the two participants remained in close proximity for the full duration of the ride, likely meant that participants felt more competent in their capabilities. However, as highlighted in the previous paragraph, this heightened perception of competence, and the associated increase in motivation to perform at a higher intensity, may be overridden by the increase in fatigue that inevitably occurs when exercising (and competing) with another person (see Parfitt et al., 2015; Park et al., 2012; Parton and Neumann, 2019).

### Hypothesis three - solo-virtual condition versus metrics-only condition

For the third hypothesis, it was predicted that the solo-virtual condition would generate higher levels of motivation compared to the metrics-only condition, and this would be associated with higher levels of effort and exertion. This hypothesis was only partially supported with interest-enjoyment and effort-importance being significantly higher for the solo-virtual condition, but there were no significant differences for tension-pressure, power, cadence, heart rate, or RPE, nor were there any differences for competence. With the exception of enjoyment, these results are inconsistent with previous research that has demonstrated the benefits of virtual exercise technology in terms of motivation and performance (e.g., Murray et al., 2016; Reason et al., 2025; see also Baños et al., 2016). However, the present results are not without exception, with other studies also failing to find benefits for virtual exercise technologies regarding either performance (e.g., Mestre et al., 2011) or perceived exertion (Baños et al., 2016; Neumann and Moffitt, 2018).

It is possible that participants in the present study approached the solo-virtual condition as a more leisurely and relaxing ride, as opposed to a performance-driven task (see also Lemmens, 2023). Indeed, the qualitative data showed that participants enjoyed the solo-virtual condition because it inferred an opportunity to cycle at their own pace and enjoy the visual information on the screen. Unlike other studies where participants were required to perform at a predetermined intensity (e.g., Mestre et al., 2011; Reason et al., 2025), the present study allowed participants to select their own pace. This sense of autonomy, combined with the relaxing features of the visual display, likely reduced their perceived need to exert additional effort, promoting a focus on enjoyment (see also Lemmens, 2023). However, it is also important to note that there were no differences between the solo-virtual and the metrics-only conditions in terms of the measures of exertion (i.e., tension-pressure, power, cadence, heart rate, and RPE). Therefore, the relaxing environment provided by the solo-virtual condition not only elicited higher levels of enjoyment, but it also produced similar levels of exertion to the metrics-only condition. It seems that exercising in a relaxing environment, where participants also have autonomy over their exercise intensity, may increase enjoyment more than a metrics-only environment, but without compromising physical exertion (see also Murray et al., 2016).

Competence was also not significantly different between the metrics-only and solo-virtual conditions, further suggesting that the impact of the second rider and the banding feature in the Zwift program could have been the primary elements that increased participants’ competence throughout the study. In real-world applications, the impact of the second rider emphasises the benefits of creating a social atmosphere where exercise can occur with others of a similar ability (see Parton and Neumann, 2019).

### Other factors influencing motivation

To further explore their perceptions regarding the future use of virtual technology as a form of indoor exercise, such as the Zwift bike used in the present study, participants were asked questions about the potential facilitators and barriers during their interviews. Twenty-five percent of participants reported that a barrier to using an indoor exercise bike was the lack of outdoor scenery and exposure to natural environmental elements (see also Liu et al., 2026; Thompson Coon et al., 2011). Additions such as fans to simulate wind, realistic outdoor routes, and ambient sounds of nature were reported by participants as features that would help to motivate them to continue using indoor exercise technology. Participants also reported accessibility being another barrier, noting that travelling long distances to use a virtual exercise device would reduce their motivation to engage with the activity (see also McDonough et al., 2020). Having a social partner with whom they could exercise was also reported as a facilitator as participants felt they would be better able to communicate more effectively indoors, compared to outdoors where environmental noise could be distracting. These findings should be interpreted with caution because the present study examined only the short-term use of the technology: examining barriers to longer-term use could produce different results.

### Limitations and future directions

This study provided valuable insights into how virtual technology and the presence of another rider influenced individuals’ motivation to ride an indoor exercise device. However, there were some limitations that should also be mentioned. First, while the use of a brief period of exercise provided valuable information on how participants initially perceived each condition, it does not accurately capture the long-term impacts on motivation, enjoyment, or exertion, nor does it show how these variables may change over time. Longitudinal studies over various time periods should be conducted to investigate whether the motivational effects of a virtual stimulus are sustained, diminished, or evolve as individuals become more familiar with the conditions (e.g., Anderson-Hanley et al., 2011; Michael and Lutteroth, 2020). These studies could determine whether the motivation experienced in the virtual conditions was due to the novelty of the activity, or whether enjoyment and engagement persist beyond the novelty period. Second, there were no measures to determine the level of competitiveness of participants. The presence of the second rider in the paired-virtual condition encouraged participants to compete, but it is possible that this may have been more pronounced in individuals with a propensity towards competitiveness, as has been shown in previous studies in this area (Anderson-Hanley et al., 2011; Snyder et al., 2012). Future research should determine the extent to which an individual’s competitive drive influences factors such as motivation and performance when exercising in the presence of others (see Parton and Neumann, 2019), and how these factors change with different performance levels (novices versus elite athletes; Neumann et al., 2018; see also Parton and Neumann, 2019) and in different situations (social exercise with a partner versus actively competing; Neumann and Moffitt, 2018).

## Conclusion

The present study revealed that the presence of a second rider when using a virtual exercise device encouraged participants to exert more effort due to a sense of competition, demonstrating the substantial influence of a second rider on an individual’s motivation to exercise (see also Anderson-Hanley et al., 2011; Nunes et al., 2014). However, as a result of this competitiveness, participants tended to over-exert themselves which resulted in a number of participants reporting that they would prefer to use a virtual exercise device by themselves, rather than alongside another person (see also Park et al., 2012). This preference stemmed from participants’ belief that they could not sustain the level of exertion required for riding with a second rider, and they would rather engage with the virtual features in a more relaxed, self-paced manner. Virtual exercise technology that did not include virtual scenery or a second rider was reported by participants as being boring and non-stimulating, highlighting how the absence of engaging elements in the visual display can reduce motivation and exertion during exercise.

## Data Availability

The datasets presented in this article are not readily available because some of the data in its raw form could compromise the anonymity of participants. Requests to access the datasets should be directed to Adam Gorman (adam.gorman@qut.edu.au).
